# Melanoma in-situ arising in seborrheic keratosis: a case report

**DOI:** 10.1186/1757-1626-1-263

**Published:** 2008-10-23

**Authors:** Susan Repertinger, Jeff Wang, Edward Adickes, Deba P Sarma

**Affiliations:** 1 Department of Pathology, Creighton University Medical Center, Omaha, NE 68131, USA

## Abstract

**Background:**

Seborrheic keratosis is a very common benign skin tumor in man. Melanoma is rare but is the most dreaded of all malignant skin tumors. A melanoma arising in a seborrheic keratosis is distinctly rare. We are reporting such a case occurring in an 86-year-old man.

**Case presentation:**

An-86-year-old male with a history of multiple actinic keratoses and seborrheic keratoses of the head and trunk presented with a mid-back skin lesion. The lesion was poorly circumscribed, flat, and gray, with a pink-tan, well-circumscribed scaly nodule within it. The biopsied lesion was composed of the usual features of hyperkeratotic seborrheic keratosis, but with focal atypical melanocytic proliferation with nesting along the dermal-epidermal junction. We interpreted this lesion as a melanoma in-situ arising within a seborrheic keratosis.

**Conclusion:**

It is not uncommon for many physicians to remove a typical seborrheic keratosis without a confirmatory microscopic confirmation. We urge that all such lesions be examined by the pathologist to avoid missing another concomitant malignant lesion such as melanoma which needs adequate resection and close follow-up.

## Background

Melanoma in-situ arising within seborrheic keratosis, while reported in the literature, is still a relatively rare entity, appearing a handful of times in the literature over the past two decades [1-4]. Seborrheic keratosis and melanoma separately are well characterized clinically and histologically. Their co-existence is of great clinical significance, given that treatment for typical appearing seborrheic keratosis may be simple destruction without histologic confirmation of the diagnosis.

## Case report

**Figure 1:**
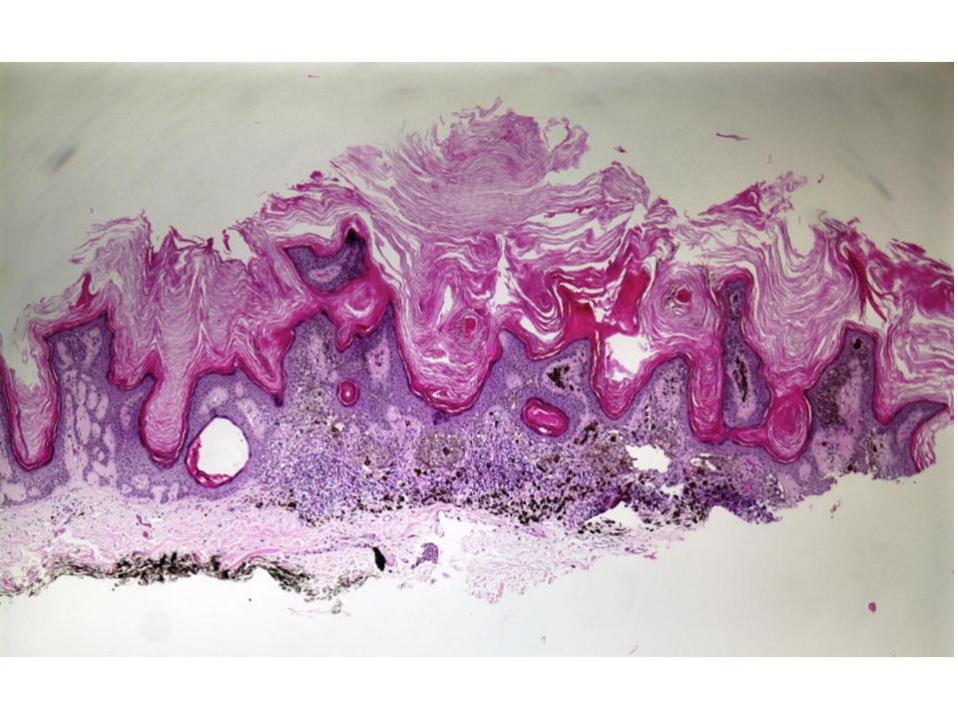
Shave biopsy of mid-back shows usual features of hyperkeratotic seborrheic keratosis with co-existing melanoma in-situ.

**Figure 2:**
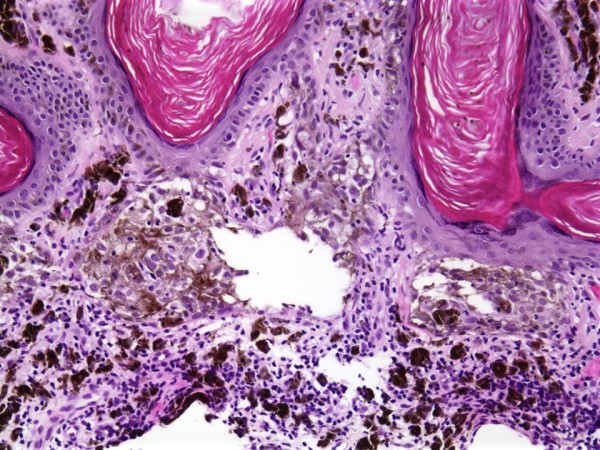
High magnification of melanoma in-situ component, with nests of atypical melanocytes along dermal-epidermal junction.

**Figure 3:**
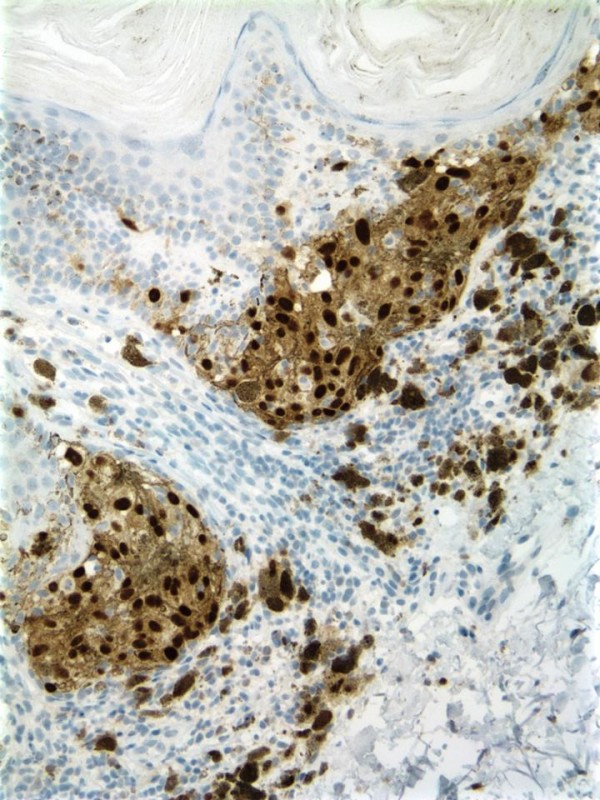
MITF immunostaining highlights malignant melanocytes.

An-86-year-old male with a history of multiple actinic keratoses and seborrheic keratoses of the head and trunk presented with a mid-back skin lesion. The lesion was poorly circumscribed, flat, and gray, with a pink-tan, well-circumscribed scaly nodule within it. The biopsied lesion was composed of the usual features of hyperkeratotic seborrheic keratosis, but with focal atypical melanocytic proliferation with nesting along the dermal-epidermal junction (Figure 1). These neoplastic melanocytes displayed pleomorphic nuclei with prominent nucleoli. There was neither any significant upward migration of the neoplastic cells, nor was there any dermal invasion. Numerous melanophages were seen within the papillary dermis [Figure 2]. The atypical melanocytes were immunoreactive with S-100, HMB-45, MART-1, and MITF (Figure 3). We interpreted this lesion as a melanoma in-situ arising within a seborrheic keratosis.

## Discussion

While the case reported here on clinical examination showed the melanocytic (gray, poorly-circumscribed) and seborrheic (scaly, well-circumscribed, nodular) components, these findings are not always evident. Therefore, histopathologic examination of all seborrheic keratoses should be considered, and is mandatory for all lesions with suspicious or changing characteristics. Lim [1] retrospectively reviewed 639 cases of seborrheic keratosis, of which 85 (9%) were found to be associated with other lesions. Of these associated lesions, 44 (7%) were malignant, with four of these arising within seborrheic keratoses. These associated lesions included premalignant lesions, malignancies, melanocytic lesions, and miscellaneous lesions.

Whether melanoma and seborrheic keratoses are coincident lesions or whether seborrheic keratosis is a precursor lesion is not clear. The fact that seborrheic keratoses are such common lesions suggests that this co-occurrence is coincidental. However, a common tumorigenic factor within the local milieu of the dermal-epidermal junction cannot be excluded as influencing the development of both lesions.

## Conclusion

This and other similar case reports underscore the importance of pathologic examination of all skin lesions, even if clinically benign. Older individuals, in particular, develop both benign and malignant skin lesions at a higher rate when compared with younger people, making co-occurrence of diagnostically disparate entities at the same skin site more likely. While the clinical appearance of the lesion in this patient suggested the presence of more than one histopathologic diagnosis, other such cases may not by clinically apparent.

## Competing interests

The authors declare that they have no competing interests.

## Authors' contributions

SR drafted the manuscript, JW prepared the photomicrographs, EA reviewed the references, and DPS conceived, revised, and submitted the manuscript. All authored have read and approved the final manuscript.

## Consent

Written consent was obtained from the patient for publication of this case report. A copy of the written consent is available for review by the Editor-in-Chief of this journal.
